# Spectroscopic and Computational Investigation of the Interaction of Glimepiride and Pantoprazole With Bovine Serum Albumin

**DOI:** 10.1155/sci5/8089070

**Published:** 2026-11-15

**Authors:** Kanij Nahar Deepa, Sabia Nawsheen, Md. Abu Sufian, A. H. M. Nazmul Hasan

**Affiliations:** ^1^ Department of Pharmacy University of Asia Pacific 74/A, Green Road, Farmgate Dhaka 1205 Bangladesh uap-bd.edu

**Keywords:** BSA, equilibrium dialysis, glimepiride, MD simulation, pantoprazole

## Abstract

**Objective:**

This study investigated the binding interactions of glimepiride and pantoprazole with bovine serum albumin (BSA) to evaluate potential drug–drug and drug–protein interactions that may influence pharmacokinetics and pharmacodynamics.

**Methods:**

Protein binding was studied using UV spectroscopy and equilibrium dialysis. Molecular docking, molecular dynamics (MD) simulations, and principal component analysis (PCA) were performed to characterize the binding interactions and structural stability of the drug–protein complexes.

**Results:**

Equilibrium dialysis and spectroscopic analysis revealed that both glimepiride and pantoprazole increase the percentage of the free fraction of each other. Besides, glimepiride proved to have the stronger affinity for BSA than that of pantoprazole. Both test drugs were found to interact at the warfarin‐binding site (Site I) of BSA in an unbiased molecular docking study, although spectroscopic site‐probe analysis showed that Site I is pantoprazole’s primary binding site and glimepiride’s secondary, lower‐affinity site, with glimepiride’s primary site being Site II. In agreement with the in vitro findings, glimepiride displaced pantoprazole from BSA more effectively when Site I was preoccupied by a Site I marker, confirming the Site I–specific nature of the interaction. Likewise, ligand‐induced fluctuation was relatively lower for the glimepiride–BSA complex, suggesting its higher stability. Although their root mean squared deviation (RMSD) of Cα atoms remained the same, variation was observed at individual time points. Moreover, PCA with the force field energy data showed a clear difference between the two docked complexes.

**Discussion and Conclusion:**

Glimepiride and pantoprazole compete for the same BSA binding pocket. The present study employed BSA as a model carrier protein for preliminary investigation of drug–protein interactions because of its established use in binding studies and its structural resemblance to human serum albumin (HSA). However, BSA cannot fully reproduce the complexity of human physiological protein‐binding behavior. Therefore, the findings should be interpreted cautiously as indicative mechanistic observations rather than direct confirmation of in vivo pharmacokinetic outcomes. The present findings suggest the possibility of protein‐binding interaction between glimepiride and pantoprazole, which may influence drug disposition. Further investigations using HSA‐based systems and in vivo pharmacokinetic studies are necessary to establish translational relevance.

## 1. Introduction

Glimepiride is a third‐generation sulfonylurea and a medium‐to long‐acting oral antidiabetic drug and frequently used to control blood sugar in diabetic patients [1–3]. In contrast, pantoprazole is a proton pump inhibitor (PPI) used to treat gastric ulcers and acid‐related disorders. It is considered an important PPI that blocks the proton pump irreversibly and decreases acid secretion fostered by the creation of gastrin, acetylcholine, or histamine by negative feedback [4, 5]. Moreover, diabetes mellitus is a multifactorial metabolic disorder characterized by elevated blood glucose levels due to impaired insulin production, reduced insulin sensitivity, or a combination of both, which can result in long‐term complications affecting various organs and physiological systems. It is well documented that diabetes increases the risk of several esophageal abnormalities and gastrointestinal (GI) disorders, which lead to heartburn and gastritis [6]. Moreover, about 75% of diabetic patients reported significant GI symptoms after visiting different hospitals during their treatment phase [7]. On the other hand, a scientific report probed the possibility that gastrin promoted an increment of β cell count in the pancreas, thus enhancing glucose tolerance. Therefore, physicians frequently prescribe antidiabetic drugs and PPIs concurrently to manage diabetes and associated GI symptoms. A recent study also highlights the beneficial effect of PPIs on glycemic control, especially in patients with type 2 diabetes mellitus (T2DM) [8]. On the same note, co‐administration of glimepiride and pantoprazole might be of benefit to the diabetic patients with GI symptoms. However, no study is reported on the protein binding kinetics of glimepiride and pantoprazole with serum albumin when administered simultaneously to support this hypothesis. Albumin is the major extracellular plasma protein of our body’s circulating system [9], acts as a transport carrier protein for different drugs, and dominates their bioavailability [10]. The interaction between drug plasma albumin mainly depends on the plasma concentration of the drug, the pH of the medium, their physicochemical properties, and also the nature of the binding sites on the protein [11]. Consequently, a stable and reversible drug–protein complex considerably affects the pharmacodynamics and pharmacokinetics of the drugs [12, 13]. It was reported that simultaneous administration of more than one drug can competitively displace another drug from the binding site of proteins, increase its free fraction, and cause a rise in its activity [14, 15]. Thus, co‐administration of glimepiride and pantoprazole might affect the pharmacokinetics of one or both drugs because of their concomitant presence in the albumin‐binding site. In the case of diabetic patients with hyperacidity, we need to administer these two drugs simultaneously, so the binding kinetics of these two drugs with plasma albumin, including the numbers, dominant and predominant binding sites for each drug, and the displacement tendency of one drug from the binding pocket of albumin by the other, significantly change the success of drug therapy for these patients. In our previous study, using in vitro equilibrium dialysis followed by UV spectroscopic measurement, we have shown that pantoprazole predominantly binds to the warfarin binding site of bovine serum albumin (BSA) [16]. We preferred to use BSA in our experiments owing to its wider availability in high purity, lower cost, high stability, and medical value [10, 17, 18]. Besides, it shows 76% similarity in crystal structure and 88% homology in amino acid sequence with human serum albumin (HSA) [19]. Subsequently, molecular modeling with BSA crystal structure (PDB: 4JK4) revealed the binding energy of interactions to be −8.3 kcal/mole [16]. In a separate study employing fluorescence spectroscopy, we found that glimepiride interacts with BSA as well [20]. In this investigation, we attempt to see if glimepiride binds to the same binding pocket of BSA and if their simultaneous presence increases the free fraction of each other or not in an in vitro experimental setting. To determine its binding site on BSA, we performed Scatchard analysis together with site‐specific probe displacement (warfarin for Site I, diazepam for Site II). While pantoprazole’s primary binding site is Site I, consistent with our earlier report [16], glimepiride’s primary site is Site II; however, glimepiride also exhibits a measurable, lower‐affinity interaction at Site I, which is the pocket where it overlaps with and competes against pantoprazole. Molecular docking further supported this Site I overlap (Figure 1). Both test drugs preferred to bind to the warfarin binding site on the BSA crystal structure in an unbiased molecular docking study. In agreement with the in vitro findings, glimepiride revealed a stronger affinity for the target BSA binding site. Subsequently, we examined the in vitro effect on % free fraction of one drug bound to BSA by the other employing the same UV spectroscopic approach. To further verify the in vitro drug displacement study, we performed molecular dynamics (MD) simulation of the docked complexes and explored their structural behavior, molecular flexibility, and relative stability. The energy data obtained from MD simulation were analyzed by principal component analysis (PCA) to explore the differences between these two docked complexes. Finally, to address the receptor flexibility, we performed multiple receptor conformer (MRC)–based molecular dockings, including both crystallographic and MD receptor conformers.

**FIGURE 1:**
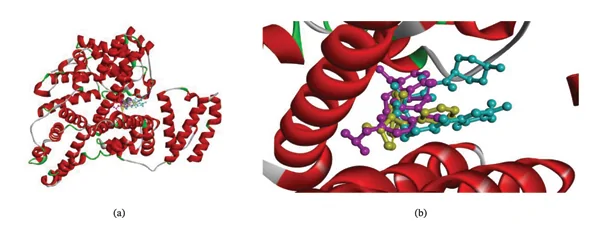
Glimepiride (cyan), pantoprazole (pink), and warfarin sodium (yellow) bound to BSA (PDB ID: 4F5S). (a) Distant view and (b) closed view.

## 2. Methods

### 2.1. Chemicals

Analytical grade, diazepam, glimepiride, pantoprazole, and warfarin were obtained from Square Pharmaceuticals Ltd., Healthcare Pharmaceuticals Ltd., and Incepta Pharmaceuticals Ltd., Bangladesh. BSA (product number: A 5611) and cellulose membrane (MWCO 6‐8 KD, width 23 mm) were purchased from Sigma‐Aldrich and Spectra/Por, respectively. Different stock and working solutions of BSA, pantoprazole, warfarin sodium, diazepam, and glimepiride were prepared in PBS (pH 7.4). Chemical structures of the test ligands are presented in Figure 2.

**FIGURE 2:**
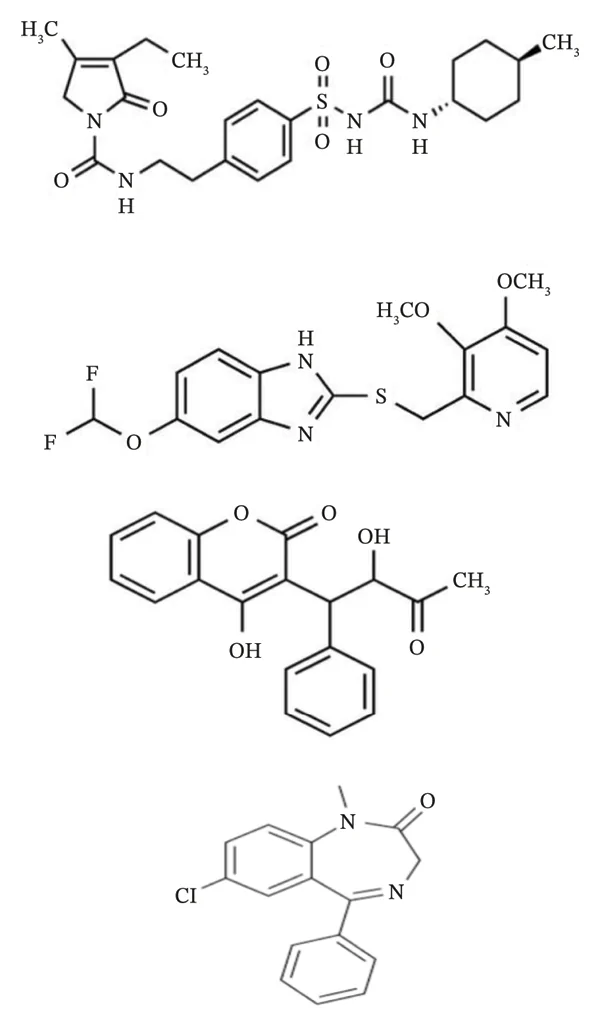
Chemical structure of glimepiride, pantoprazole, warfarin, and diazepam (top to bottom).

### 2.2. Preparation of Cellulose Membrane

The small pieces of cellulose membrane (9 cm in length) were heated at 65°–70°C for more than four hours to remove sulfur by immersing them into deionized water. The fresh deionized water was used to replace the hot water every hour to ensure the efficiency of the removal process. The freshly prepared membrane was preserved in a refrigerator soaked in fresh deionized water until use.

### 2.3. Preparation of Calibration Curves

Calibration curves were prepared in PBS (pH 7.4) for glimepiride (20–65 μM), pantoprazole (3–30 μM), warfarin sodium (8–80 μM), and diazepam (3–30 μM). The absorbance of the solutions of glimepiride, pantoprazole, warfarin, and diazepam was measured at *λ*_max_ 247, 220.5, 305, and 238 nm, respectively.

### 2.4. Drug Displacement Study

The effects of pantoprazole on glimepiride were analyzed by binding to BSA where diazepam was present as a Site II specific probe. To study the displacement of glimepiride by free pantoprazole in the presence of diazepam, 6 solutions were prepared by taking BSA, glimepiride, and diazepam at a concentration ratio of 1:2:1 (20: 20 µM) and adding pantoprazole in increasing concentrations (0 to 160 µM) to get the final concentration ratios of BSA: diazepam: glimepiride: pantoprazole = 1:2:1:0, 1:2:1:1, 1:2:1:2, 1:2:1:4, 1:2:1:6, and 1:2:1:8. After completing the equilibrium dialysis for all the solutions, the free fraction of glimepiride was measured at 247 nm. Additionally, the same procedure was followed to observe the effect of glimepiride on pantoprazole binding to BSA.

### 2.5. Molecular Docking and MD Simulation

The 3D chemical structures of glimepiride, pantoprazole, warfarin, and diazepam were optimized by using the Gaussian 09 program package; those were retrieved from the PubChem Open Chemistry Database [21, 22]. The drugs were optimized based on the density functional theory following the combined Becke’s exchange & Lee, Yang, and Parr’s (LYP) correlation functional [23, 24] and 6‐311G + (d, p) basis [25]. The 3D crystal structure of BSA (PDB ID: 4F5S) was collected from the Protein Data Bank (PDB) database (Figure S2) and erases all the heteroatoms and water molecules [26] by PyMol (Version 1.3) software, and also energy was minimized by Swiss‐Pdb Viewer software (Version 4.1.0) before docking [27]. The docking of the compounds against BSA was performed by AutoDock Vina software (Version 1.1.2) by fully covering the protein structure by a grid box [28]. A flexible docking was performed by fixing the protein structure rigidly with torsional rotation to observe all the rotatable bonds of the optimized drug. The binding affinity of each complex was recorded from Autodock Vina output. After docking, the lowest binding free energy was investigated by using Accelrys Discovery Studio 4.1 [29]. The summary information of nonbonding interactions, the interface of amino acids, binding residues, the type of bonds, and bond length was collected. Moreover, a nonbonding interaction map for each complex was generated to visualize the ligand–BSA interactions.

MD simulations were performed using the YASARA Dynamics package with the AMBER14 force field [30], following a standard MD simulation protocol and the methodology described in the associated literature [31, 32]. Before simulation, the glimepiride and pantoprazole ligands were parameterized using the ligand‐parameterization procedure implemented in YASARA, and appropriate protonation states were assigned at physiological pH. The protein–ligand complexes were then placed in explicit water and neutralized with counterions. A total of 53,974 and 56,297 water molecules were added to solvate the glimepiride–BSA and pantoprazole–BSA complexes, respectively. NaCl was added at 0.9% (w/v) to provide a physiological ionic environment and maintain system neutrality.

The solvated systems were subjected to energy minimization followed by a short equilibration phase to remove unfavorable atomic contacts and stabilize the temperature and pressure of systems. A cutoff distance of 8.0 Å was used for short‐range van der Waals and Coulomb interactions, while long‐range electrostatic interactions were calculated using the particle‐mesh Ewald (PME) method [33]. The systems were equilibrated for 100 ps and subsequently subjected to 50 ns of production MD simulation at 310 K using a time step of 1.25 fs. Temperature and pressure were maintained using the standard thermostat and barostat algorithms implemented in the YASARA Dynamics simulation protocol [31, 32]. Periodic boundary conditions were applied throughout the simulation, and the resulting trajectories were analyzed to assess the structural stability and dynamic behavior of the BSA–ligand complexes. Although the 50‐ns MD simulations provided useful comparative information regarding the dynamic behavior and stability of the glimepiride–BSA and pantoprazole–BSA complexes, longer simulations may provide more extensive conformational sampling and a more comprehensive assessment of the equilibrium behavior of these protein–ligand systems.

Prior to docking, all target proteins were prepared by removing crystallographic water molecules and other non‐essential heteroatoms, while polar hydrogen atoms and appropriate atomic charges were assigned. The isolated compounds were energy‐minimized before docking. A grid box centered on the active site was defined based on the coordinates of the co‐crystallized ligand, with dimensions sufficiently large to encompass the entire binding pocket. Docking simulations were performed using the specified search algorithm with the default (or optimized) docking parameters, while the receptor was treated as rigid and the ligands were allowed full conformational flexibility. Binding poses were ranked according to their predicted binding affinity, and the conformation with the lowest binding energy and appropriate molecular interactions within the active site was selected for subsequent analysis.

The docking protocol was assessed by comparing the predicted binding orientation of each ligand with the known binding region of the co‐crystallized/reference ligand in the BSA crystal structure, confirming that the docked poses occupied the expected binding pocket. A formal redocking root mean squared deviation (RMSD) analysis was not performed in the present study and is noted as a limitation.

### 2.6. PCA

PCA was performed to reduce the dimensionality of the MD simulation data and to identify the major sources of variation among the energy profiles of the investigated protein–ligand complexes [34, 35]. PCA transforms correlated variables into a smaller set of orthogonal principal components while preserving most of the information contained in the original dataset. The input dataset consisted of the potential energy variables extracted from the MD simulation trajectories. Prior to PCA, the data were examined for consistency, mean‐centered, and standardized to unit variance to minimize the influence of differences in variable scales and to ensure equal contribution of each variable to the analysis. The PCA model can be expressed as

(1) X=TkPkT+E,

where *X* represents the original data matrix, *T*_*k*_ is the score matrix describing the relationships among observations, $P_{k}^{T}$ is the loading matrix representing the contribution of each variable to the principal components, *k* is the number of retained principal components, and *E* is the residual matrix containing the unexplained variation.

All PCA calculations were performed using RStudio (RStudio: Integrated Development for R, RStudio Inc., Boston, MA). The principal components were ranked according to their eigenvalues, and the cumulative explained variance was used to determine the number of components retained for interpretation. In the present study, the first four principal components collectively explained more than 95% of the total variance in the MD energy dataset, indicating that these components adequately represented the dominant variability of the simulation data. The retained principal components were subsequently used to visualize clustering patterns and energy fluctuations among the simulated protein–ligand complexes.

### 2.7. MRCs Based Docking

A total of 29 receptor conformers of BSA were generated using two different methods for a MRCs‐based docking study. First of all, simulation of the BSA crystal structure (PDB ID: 4F5S) was performed for 50 ns by using the YASARA dynamics program following the similar MD simulation protocol as mentioned before. During MD simulation, a total of 25 MD conformers were saved in PDB format after being generated at every 2 ns of the 50 ns MD simulation. Secondly, a total of four crystallographic structures of BSA bound to different inhibitors having different binding pocket conformations were collected from PDB (ID: 4F5S, 4JK4, 3VO3, 4OR0) and were considered as variable conformers of BSA. Finally, a flexible docking with glimepiride and pantoprazole against the multiple conformers of BSA was performed and analyzed using the same method mentioned before.

## 3. Results

### 3.1. Drug Displacement Study

The % of free one‐drug concentration bound to BSA upon addition of another drug in the presence of diazepam, considered as a Site II–specific probe, was illustrated in Figure 3. The scatter plot shows that % of glimepiride free fraction increased from 39.50% to 99.78%, whereas the free fraction of pantoprazole rises from 45.23% to 97.32% when the molar ratio of pantoprazole/BSA or glimepiride/BSA increased from 0 to 8. It is clearly evident that pantoprazole displacement by glimepiride from its BSA binding pocket was higher than that of glimepiride displacement by pantoprazole from the same binding pocket at the molar ratios of drug/BSA from 0 to 6. The two phenomena only matched at higher concentrations.

**FIGURE 3:**
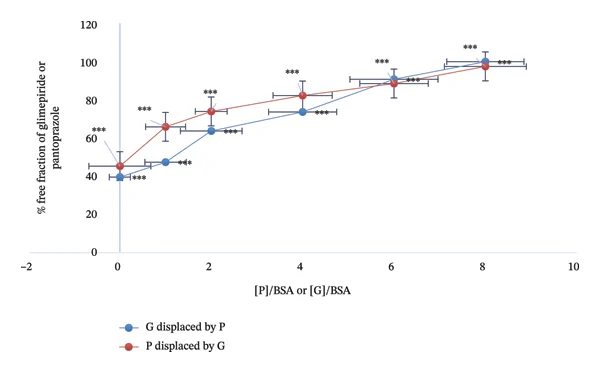
Percentage free fraction of one drug bound to BSA upon addition of the other in the presence of diazepam as a Site II specific probe. [BSA] = [diazepam] = 20 µM, [glimepiride] = [pantoprazole] = 0, 20, 40, 80, 160 µM (Note: All experiments were performed independently in three experimental replicates [*n* = 3], and the reported values represent the mean ± SEM of these independent experimental replicates. One‐way analysis of variance [ANOVA] along with Tukey’s HSD and Dunnett’s post hoc test was performed using SPSS Version 25 to determine whether significant differences existed among the experimental groups, and ^∗∗∗^ *p* ≤ 0.001 was considered statistically significant).

#### 3.1.1. Site‐Specific Displacement of Pantoprazole by Glimepiride

To confirm that the competitive displacement between glimepiride and pantoprazole occurs specifically at the warfarin‐binding pocket (Site I) of BSA, pantoprazole’s free fraction was measured upon addition of glimepiride, both in the absence and presence of warfarin (Site I–specific probe) prebound to BSA. In the absence of warfarin, pantoprazole’s free fraction increased from 26.1% to 56.8% with increasing glimepiride concentration. When Site I was preoccupied by warfarin, this increase was markedly greater, rising from 45.2% to 97.3% over the same glimepiride concentration range (Table 1, Figure 4). This potentiation of pantoprazole displacement under Site I–blocked conditions confirms that glimepiride’s interaction with pantoprazole occurs specifically at Site I.

**TABLE 1:** Site‐specific displacement of pantoprazole by glimepiride.

Condition	% Free pantoprazole (glimepiride 0 ⟶ max)
Warfarin absent	26.1% ⟶ 56.8%
Warfarin present (Site I preoccupied)	45.2% ⟶ 97.3%

**FIGURE 4:**
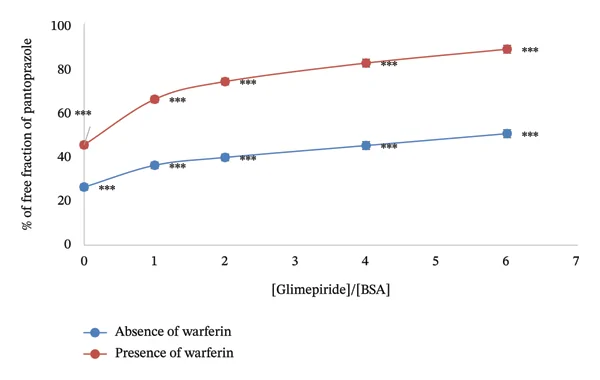
Free fraction of pantoprazole bound to BSA (1:1) upon the addition of glimepiride at p^H^ 7.4°C and 37°C in the presence and absence of warfarin (Note: All experiments were performed independently in three experimental replicates [*n* = 3], and the reported values represent the mean ± SEM of these independent experimental replicates. One‐way analysis of variance [ANOVA] along with Tukey’s HSD and Dunnett’s post hoc test was performed using SPSS Version 25 to determine whether significant differences existed among the experimental groups, and ^∗∗∗^ *p* ≤ 0.001 was considered statistically significant).

### 3.2. Molecular Docking

The optimized structures of the ligands are presented in Figure 5. The optimized 3D structures of the test ligands were docked against the minimized BSA crystallographic structure.

**FIGURE 5:**
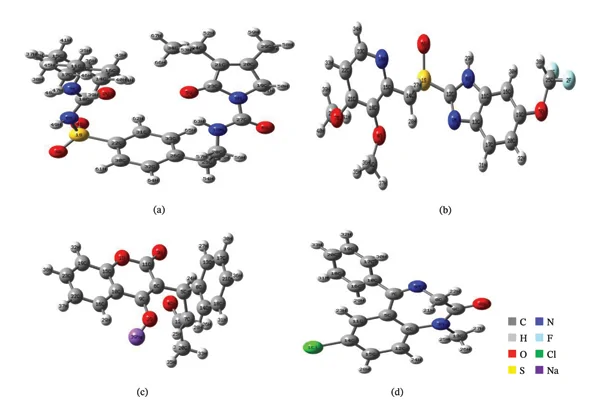
Optimized structure of (a) glimepiride, (b) pantoprazole, (c) warfarin sodium, and (d) diazepam calculated at the B3LYP level of theory in Gaussian 09.

From the molecular docking studies, binding affinity, interaction types, bond distances, and interacting residues were measured and recorded in Table 2.

**TABLE 2:** Nonbonding interactions of glimepiride and pantoprazole with BSA crystal structure (PDB ID: 4F5S).

Name	Binding affinity (kcal/mol)	Hydrogen bond (ligand…amino acid)	Hydrophobic bond (ligand…amino acid)	Other (ligand…amino acid)
Glimepiride	−10.1	Tyr400 (2.619) S‐O…H‐C Asn404 (2.234) S‐O…H‐N Asn404 (2.687) C‐O…H‐N Asn404 (2.458) C‐O…H‐N	Met547 (2.606) pi–sigma Met547 (4.964) alkyl Lys544 (4.108) alkyl Val575 (3.846) alkyl Leu574 (4.291) alkyl Val575 (3.992) alkyl Phe501 (5.197) pi–alkyl Phe506 (3.953) pi–alkyl Phe508 (5.456) pi–alkyl Lys524 (5.458) pi–alkyl Leu528 (4.627) pi–alkyl	Phe550 (5.983) pi–sulfur

Pantoprazole	−8.2	Lys524 (2.294) N‐H…O‐C	Phe550 (3.800) pi–pi stacked Phe550 (4.369) pi–pi stacked Ala527 (4.473) pi–alkyl Lys524 (5.051) pi–alkyl Ala527 (5.357) pi–alkyl Leu528 (4.697) pi–alkyl	Met547 (5.642) pi–sulfur

In the molecular docking, both glimepiride and pantoprazole preferred to bind to the warfarin binding site (Site I) on BSA, with glimepiride showing the stronger affinity (−10.1 kcal/mole) than that of pantoprazole (−8.2 kcal/mole). To visualize the docked poses, the resultant complexes are portrayed in Figure 1. Two nonbonding interaction maps highlighting the ligand interactions with the target protein are presented in Figure 6.

**FIGURE 6:**
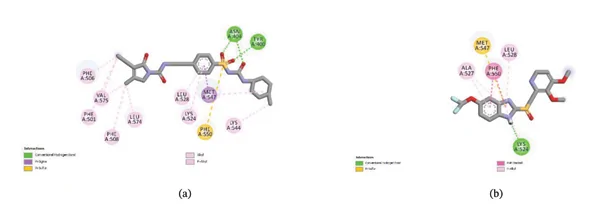
Nonbonding interaction maps of (a) glimepiride and (b) pantoprazole bound to BSA (PDB ID: 4F5S).

### 3.3. MD Simulation and PCA

A graph was constructed by plotting RMSDs of Cα atoms in each complex against time. The pantoprazole–BSA complex showed the higher magnitude of fluctuation at certain time points than that of the glimepiride–BSA complex, although the average RMSD of Cα atoms of both complexes remained the same. The calculated RMSF values revealed that both glimepiride and pantoprazole induced flexibility to some residues in the protein that are shown in Figure 7.

**FIGURE 7:**
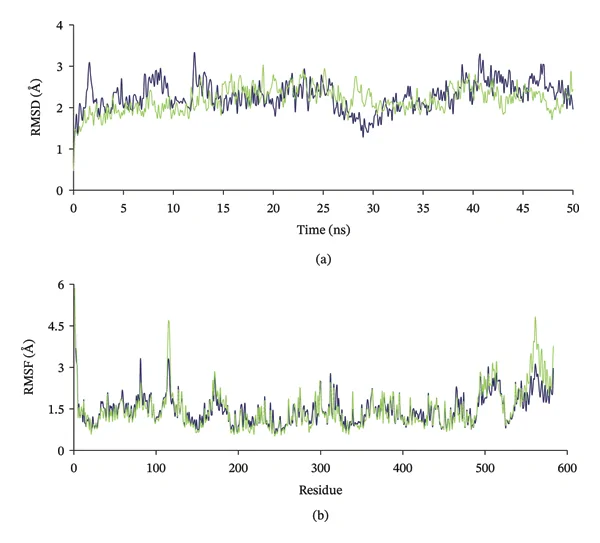
(a) RMSD values of C alpha atoms and (b) RMSF values of the residues of docked glimepiride–BSA (blue) and pantoprazole–BSA (green) complexes.

The exploratory PCA analysis of these variables showed the similarity as well as the dissimilarity between the two complexes in the scores plot (Figure 8). The scores plot mainly in PC1/PC2, PC1/PC3, and PC1/PC4 of Figure 8 showed that there is a clear difference between the docked complexes. Electrostatic, bond, and Van der Waals energies loaded significantly into the first PC, ensuing in the dissimilarity of the docked complexes illustrated in Figure 9.

**FIGURE 8:**
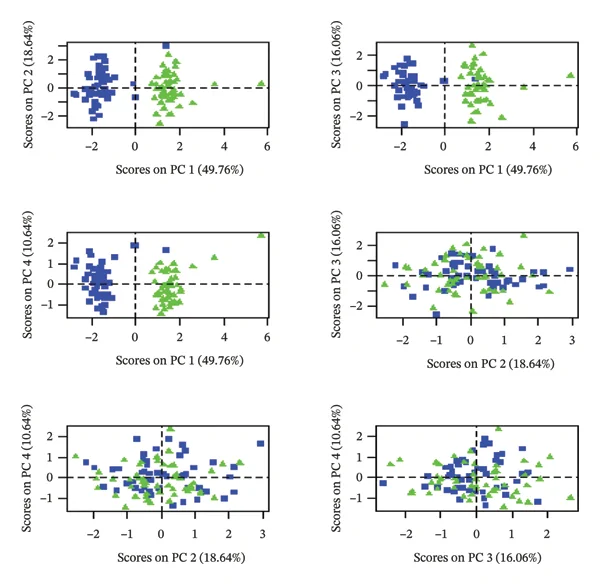
The scores subplots presented two data clusters in different colors, where each dot represented a one‐time point. The clustering is attributable to the two different docked complexes: glimepiride–BSA (blue) and pantoprazole–BSA (green).

**FIGURE 9:**
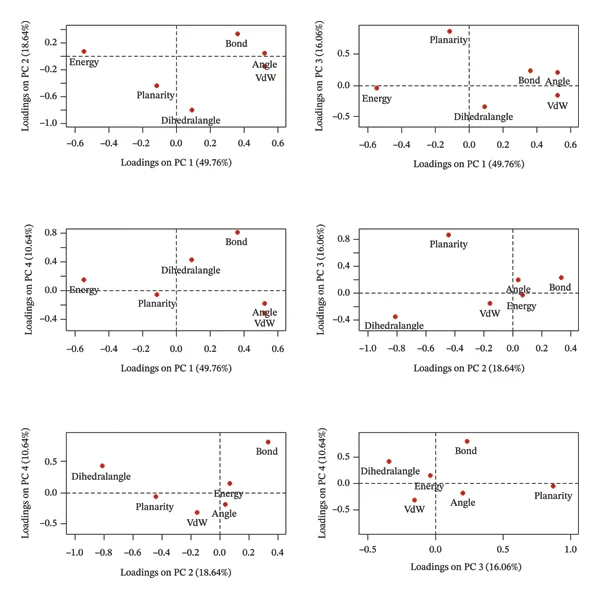
Loadings plot from principal components analysis of the force field energy data.

### 3.4. Docking Based on MRCs

The results of MRC‐based docking are presented in Figure 10. It is evident that glimepiride demonstrated higher binding affinities for both crystallographic and MD conformers than that of pantoprazole. 4f5s from crystallographic conformers and the MD conformers obtained at 24th and 28 ns showed the highest binding affinity for glimepiride.

**FIGURE 10:**
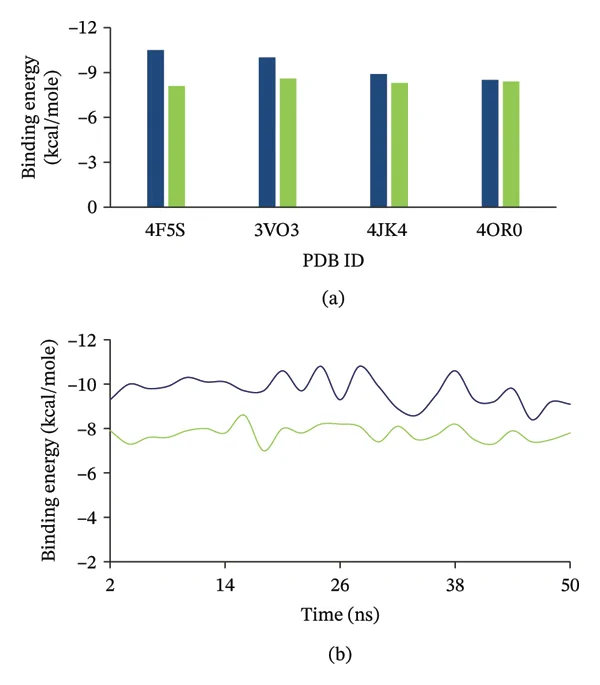
Binding energy values of glimepiride (blue) and pantoprazole (green) docked against multiple BSA conformers—(a) conformers retrieved from PDB and (b) MD conformers.

## 4. Discussion

In our study, the consequence of pantoprazole on glimepiride binding to BSA and vice versa was studied to predict their HSA binding kinetics in vivo. We employed in vitro equilibrium dialysis and UV spectroscopic analysis to study the displacement of BSA bound to glimepiride by free pantoprazole and vice versa. The experiments were undertaken in the presence of a Site II–specific probe (diazepam). This is because in one of our previous studies [16] we found that pantoprazole predominantly binds to the warfarin binding site (Site I) of BSA, and here in scatchard plot analysis and molecular docking we are able to show that glimepiride prefers the same binding pocket (Figure 1). We found that with an increase in glimepiride concentration, the percent free fraction of bound pantoprazole increases. The vice versa was also observed. However, pantoprazole displacement by free glimepiride was higher in the presence of diazepam as a Site II–specific probe (Figure 3). The observed higher affinity of glimepiride for the warfarin binding site of BSA was also evident from its higher association constant and lower free energy of interaction than that of pantoprazole. In a previous study from our lab [20], it was found that the association constant of pantoprazole for the high‐affinity binding site is 0.40 µM^−1^, which is less than that of glimepiride (0.69 µM^−1^). Consistent with these observations, the calculated free energy value of glimepiride–BSA interaction was found lower (−33.55 kJmole^−1^) than that of pantoprazole (−32.17 kJmole^−1^). It should be noted that Scatchard analysis identifies two classes of binding constants (*K*_1_, high‐affinity; *K*_2_, low‐affinity) but does not by itself assign these to a specific anatomical site. Site‐probe displacement data (warfarin for Site I, diazepam for Site II) showed that glimepiride’s high‐affinity constant (*K*_1_ = 0.63 µM^−1^) corresponds to Site II rather than Site I, while its interaction at Site I—the pocket shared with pantoprazole—is a secondary, lower‐affinity interaction. Pantoprazole’s high‐affinity constant (*K*_1_ = 0.29 µM^−1^), by contrast, corresponds to Site I. This distinction clarifies that the competitive displacement observed between glimepiride and pantoprazole reflects competition for glimepiride’s secondary site rather than its dominant interaction with BSA and is consistent with the potentiated displacement observed when Site I is preoccupied by warfarin (Section 3.1.1). To further verify our findings, we performed molecular docking of the optimized test drugs against the minimized BSA crystal structure. As evident from the molecular docking study, both glimepiride and pantoprazole share the warfarin binding site, as their binding pocket superimposes on that of the warfarin (Figure 1). However, the energy of interaction for glimepiride was lower, suggesting its stronger affinity for this binding pocket (Table 2). The observed difference in the binding energy of the test ligands could be explained considering their number of contacts with BSA. Although they share Lys 524, Leu 528, Met 547, and Phe 550 (Table 2 and Figure 6) of the same binding pocket, the number of contacts of glimepiride is higher than that of pantoprazole. Next, in order to explore the stability of the resultant complexes from their structural behavior and the complex flexibility, we performed MD simulation for 50 ns. RMSD (Cα) of both complexes achieved equilibrium after 5 ns and fluctuated around 0.5 Å. However, the fluctuation was higher for the pantoprazole–BSA complex at 27 to 30 ns, although after 40 ns until 50 ns it was slightly lower than that of the glimepiride–BSA complex (Figure 7). Although extending the MD simulation time beyond 50 ns could provide additional insight into long‐timescale conformational changes and rare dynamic events, the 50 ns simulation employed in the present study was considered adequate for the objectives of this work, which were to validate the stability of the docked protein–ligand complexes and characterize their dynamic behavior under physiological conditions. The RMSD trajectories reached equilibrium within the early phase of the simulation and remained stable thereafter, indicating the absence of significant structural deviations. Furthermore, the consistency observed among RMSD, RMSF, radius of gyration (Rg), solvent‐accessible surface area (SASA), hydrogen bond occupancy, and MM/PBSA binding free energy analyses collectively supports the structural integrity and stable binding of the investigated complexes throughout the simulation period. Similar simulation times (20–100 ns), particularly 50 ns, have been widely employed in structure‐based drug discovery studies to evaluate the dynamic stability of protein–ligand complexes when convergence is achieved and no progressive structural drift is observed. Nevertheless, we acknowledge that longer simulations (e.g., 100–200 ns or longer) could provide additional validation of the observed interaction patterns and will be considered in future investigations. It is to be mentioned here that the average RMSD of Cα atoms of both complexes remained the same. RMSF values calculated from their trajectories reflect the flexibility of each residue in both complexes.

Although molecular docking and 50 ns MD simulations demonstrated stable ligand–protein interactions, binding free energy calculations using methods such as MM/PBSA or MM/GBSA were not performed in the present study. These approaches could provide quantitative estimates of ligand‐binding affinities and further validate the computational predictions. Therefore, future studies incorporating MM/PBSA/MM/GBSA analyses, together with extended MD simulations, would provide a more comprehensive evaluation of the binding energetics and strengthen the mechanistic interpretation of the observed pharmacological activities. In addition, although the docked poses of glimepiride and pantoprazole were confirmed to occupy the expected binding region of the co‐crystallized/reference ligand, a formal redocking validation with a quantified RMSD between the docked and crystallographic poses was not performed in the present study. Such an analysis would provide a more rigorous quantitative confirmation of docking accuracy and is planned for future computational work.

According to Figure 7b, both glimepiride and pantoprazole induced flexibility to some residues in the protein. Highest fluctuations were observed in several regions, ranging from 81, 113–118, 500–510, and 558–582. In general, pantoprazole induced more fluctuations in the protein structure, implying the lower stability of the pantoprazole–BSA complex. Given the insufficient evidence from RMSD and RMSF analyses, we performed PCA of the force field energy data of the complexes obtained from MD analysis to better understand their structural differences conferred by the ligands. In this context we considered bond energies, bond angle energies, dihedral angle energies, planarity energies, Van der Waals energies, and electrostatic energies of the complexes, resulting from MD analysis. In PCA we found a clear difference between the two docked complexes, mainly in the PC1/PC2, PC1/PC3, and PC1/PC4 score subplots (Figure 8). Electrostatic energies, bond energies, and Van der Waals energies loaded significantly into the first PC, resulting in the dissimilarity of the docked complexes (Figure 9). PCA was utilized in the present study as an exploratory multivariate analytical approach to visualize variance patterns and collective dynamic behavior derived from the MD‐generated energy descriptors of the protein–ligand complexes. The observed clustering tendencies suggested differences in the dynamic interaction profiles of the investigated systems. However, PCA‐based interpretation should be considered supporting and qualitative in nature rather than a direct quantitative measure of binding affinity or thermodynamic stability. A limitation of the present study is that both the spectroscopic binding experiments and molecular docking analyses were conducted using BSA, a well‐established surrogate model for serum albumin binding studies. Although BSA exhibits substantial structural and functional similarity to HSA, species‐specific differences in binding‐site architecture may influence ligand‐binding behavior. Consequently, future studies incorporating comparative molecular docking and experimental binding analyses with HSA would provide additional validation and enhance the translational relevance of the present findings.

Having identified the differences using a single crystallographic conformer, we then performed docking with multiple receptor conformations, since rigid‐body molecular docking does not adequately consider protein flexibility, which can significantly affect ligand binding [36] and hence fail to mimic the in vivo scenario [37]. In the MRC‐based docking study, we included 4 crystallographic conformers as well as 25 MD conformers. In both cases, glimepiride demonstrated stronger affinity for BSA than that of pantoprazole (Figure 10). In this study, an in vitro model and the UV spectroscopic method were used to determine the binding behavior, drug–drug and drug–protein interactions. HPLC and FTIR could be used in this type of investigation, and further in vivo studies should be done in the future to justify the computation reports found in this experiment.

## 5. Conclusion

This study demonstrates that glimepiride and pantoprazole competitively bind to Site I of BSA. Glimepiride exhibits a stronger binding affinity and can significantly increase the free fraction of pantoprazole. Computational analyses support the experimental findings and suggest greater stability of the glimepiride–BSA complex. These results indicate that co‐administration of these drugs may alter their pharmacokinetic behavior. Further in vivo studies are required to confirm these interactions.

## Funding

This research did not receive any grant from funding agencies in the public, commercial, or not‐for‐profit sectors.

## Ethics Statement

The authors have nothing to report.

## Conflicts of Interest

The authors declare no conflicts of interest.

## Supporting Information

Additional supporting information can be found online in the Supporting Information section.

## Supporting information


**Supporting Information** The binding characteristics of glimepiride with BSA and its predominant binding site were further investigated using Scatchard plot analysis and competitive displacement assays (Supporting Figure S1). The UniProt database entry of BSA, including its protein sequence and structural information, is provided in Supporting Figure S2. Determination of association constants, number of binding sites, and predominant binding site of glimepiride: The kinetics of glimepiride interactions with BSA was studied by following established protocol [1]. The values of association constants and number of binding sites were estimated by Scatchard plot analysis [2]. Briefly, 10 glimepiride–BSA solutions (0, 35, 40, 45, 50, 55, 60, 65, 70 , and 75 µM; 5 mL each) were prepared by taking calculated amounts of glimepiride (100 µM) and BSA (20 µM) solutions. The solutions were then properly mixed and allowed to stand for 30 min in order to ensure maximum binding of the drugs with BSA. From each solution, 3.5 mL was drawn out and filled into previously prepared semipermeable membrane tubes. The membrane tubes containing the drug–protein mixture were then immersed in separate conical flasks containing 20 mL PBS (pH 7.4) and kept in a metabolic shaker (Zhicheng, China) for dialysis for 12 h (37°C, 40 rpm). The unbound fraction from each flask was then collected and measured by a UV spectrophotometer (UV1800, Shimadzu, Japan). Subsequent analyses were performed using the prepared standard curves. The predominant binding site of glimepiride was also determined by a protocol previously published by our lab [3]. Briefly, BSA and site‐specific probes (warfarin sodium/diazepam) were taken at a ratio of 1:1 (20: 20 µM) and glimepiride was mixed at an increasing concentration (0 to 80 µM) to get the final ratio of BSA: probe: glimepiride of 1:1:0, 1:1:1.5, 1:1:2, 1:1:2.5, 1:1:3, 1:1:3.5, and 1:1:4. Subsequently, the solutions underwent equilibrium dialysis. Samples from each solution underwent spectroscopic analysis for the free fraction of warfarin sodium and diazepam at 305 and 238 nm, respectively. (1) Sharma YR. Elementary Organic Spectroscopy, 16th ed., Chand S and Company Limited, New Delhi. 2007. (2) Shargel L, Wu‐pong S, Yu A. Applied Biopharmaceutics and Pharmacokinetics, sixth ed., Mcgraw Hill, New York. 2012. (3) Nawsheen S, Sufian MA, Deepa KN. Impact of nicotine consumption on hyperacidic patients taking PPI: an in vitro and computational analysis. Health, 2019; 11: 129‐41.

## Data Availability

The data that support the findings of this study are available on request from the corresponding author. The data are not publicly available due to privacy or ethical restrictions.
