# Supplementary material for: Spectroscopic and Computational Investigation of the Interaction of Glimepiride and Pantoprazole With Bovine Serum Albumin
Source: Scientifica (Cairo). 2026 Nov 15;2026:8089070. doi: 10.1155/sci5/8089070 (PMC13770319; doi:10.1155/sci5/8089070)
Supplement: Supplementary file 1 [file SCI5-2026-8089070-s001.docx]

**SUPPLEMENTARY INFORMATION**

**Spectroscopic and Computational Investigation to Probe the Interactions of Glimepiride and Pantoprazole with Bovine Serum Albumin**

| r | r/[Df] |
| --- | --- |
| 0.520922 | 0.227399 |
| 0.614894 | 0.159669 |
| 0.680496 | 0.119489 |
| 0.724823 | 0.0935041 |
| 0.790426 | 0.0823725 |
| 0.856028 | 0.0748295 |
| 0.914539 | 0.0684811 |
| 0.965957 | 0.0629681 |
| 1.01738 | 0.0587188 |

a)

% Pf

| [Glimepiride]/[BSA] | Warfarin sodium | Diazepam |
| --- | --- | --- |
| 0 | 14.15 | 7.49245 |
| 1.5 | 15.65 | 7.79456 |
| 2 | 19.65 | 8.70091 |
| 2.5 | 27.15 | 10.5136 |
| 3 | 39.15 | 13.2326 |
| 3.5 | 61.65 | 17.1601 |
| 4 | 100.15 | 22.9003 |

b)

**Figure S1**: a) Scatchard plot of glimepiride bound to bovine serum albumin at p^H^ 7.4 and 37 ºC. r = [bound drug]/[BSA], [D_f_ ] = free drug conc., [BSA] = 20 µM, [glimepiride] = 15-55 µM (n_1,_ n_2,_ k_1_ and k_2_ = 0.87, 1.5, 0.69 µM^-1^ and 0.133 µM^-1^, respectively), b) % free fraction of probe (% P_f_) vs [drug]/[BSA] graph. [BSA] = [diazepam] = [warfarin sodium] = 20 µM, [glimepiride] = 0, 30, 40, 50, 60, 70 and 80 µM

**Figure S2**: UniProt entry of bovine serum albumin
